# The Resurgence of Medical Education in Sociology: A Return to Our Roots and an Agenda for the Future

**DOI:** 10.1177/0022146521996275

**Published:** 2021-09-16

**Authors:** Tania M. Jenkins, Kelly Underman, Alexandra H. Vinson, Lauren D. Olsen, Laura E. Hirshfield

**Affiliations:** 1 The University of North Carolina at Chapel Hill, Chapel Hill, NC, USA; 2 Drexel University, Philadelphia, PA, USA; 3 University of Michigan Medical School, Ann Arbor, MI, USA; 4 Temple University, Philadelphia, PA, USA; 5 University of Illinois College of Medicine, Chicago, IL, USA

**Keywords:** medical education, medical profession, medical residents, medical students, socialization

## Abstract

From 1940 to 1980, studies of medical education were foundational to sociology, but attention shifted away from medical training in the late 1980s. Recently, there has been a marked return to this once pivotal topic, reflecting new questions and stakes. This article traces this resurgence by reviewing recent substantive research trends and setting the agenda for future research. We summarize four current research foci that reflect and critically map onto earlier projects in this subfield while driving theoretical development elsewhere in the larger discipline: (1) professional socialization, (2) knowledge regimes, (3) stratification within the profession, and (4) sociology of the field of medical education. We then offer six potential future directions where more research is needed: (1) inequalities in medical education, (2) socialization across the life course and new institutional forms of gatekeeping, (3) provider well-being, (4) globalization, (5) medical education as knowledge-based work, and (6) effects of the COVID-19 pandemic.

From the 1940s to 1980s, studies of medical education were foundational to sociology, with works like *The Student Physician* (Merton, Reader, and Kendall 1957), *Boys in White* (Becker et al. 1961), and *Forgive and Remember* (Bosk 1979) becoming classics in not only medical sociology but also the broader sociological canon. Such studies of medical education informed emerging sociological literatures on socialization, the professions, and social control.

However, attention shifted away from medical training in the late 1980s for several reasons. Bloom (2002) chronicled the challenges sociologists experienced working in medical schools, which resulted in greater restrictions on sociologists’ access to medical training contexts. Central to these difficulties was a conflict over whether sociological research should serve the concerns of the medical field (sociology *in* medicine) or critically interrogate medical training and work (sociology *of* medicine; Straus 1957). Rising interest in structural changes in health care and transformations in the way sociologists conceptualize professions further drew sociologists away from these contexts (Vinson 2015). Research on illness experience, technological and financial transformations, the social organization of scientific knowledge, social determinants of health, and countervailing powers became more common among medical sociologists during this time. Although influential work was published on the hidden curriculum and emotional socialization in the 1980s (Anspach 1988; Hafferty 1988; Light 1988), sociological attention to medical education was limited.

Recently, there has been a marked return to this once pivotal topic. In the past two decades, studies of medical education have examined broad transformations in medicine that have altered what it means to become a physician in the 21st century. These transformations include the rise of patient consumerism, evidence-based medicine, and the pharmaceutical industry (Timmermans and Oh 2010). Today’s medical students look quite different than the “boys in white”: They come from more diverse backgrounds, have more types of knowledge and skills to master, and experience more overt forms of socialization (Underman and Hirshfield 2016). Recent studies of medical education reflect new contexts, questions, and stakes. Indeed, since 2000, more than 200 peer-reviewed articles and books, one handbook, and at least two conferences have focused on the sociology of medical education. This subfield is once again burgeoning, with important implications for broader sociology.

In this article, we trace the resurgence of the sociology of medical education by reviewing research trends from the past 20 years, illustrating the continuities and discontinuities with previous scholarship as well as contributions to the broader discipline while setting the agenda for future research. For reasons of scope, we focused exclusively on the sociology of medical education, which meant excluding excellent work on other health professions as well as research by physicians, anthropologists, and others investigating medical training. We designed our literature search to use databases most representative of sociological and medical education journals: Sociological Abstracts and PubMed. Using keyword searches of the MeSH terms “education, medical,” and “sociology affiliation AND medical education OR Education” from 2000 to 2020, as well as our own knowledge of the field, we assembled more than 200 peer-reviewed articles and books. For works to be included in our review, at least one author had to be a trained sociologist or appointed in a sociology department, and the topic of the article, its methods, or its theories had to draw on or contribute to sociological work. We compiled these references and collaboratively identified four major research themes: (1) professional socialization, (2) knowledge regimes, (3) stratification within the profession, and (4) sociology of the field of medical education. In reviewing these themes, we find that recent work in the sociology of medical education has not only extended the three original areas of sociology pioneered by the classics—socialization, professions, and social control—but has also drawn from subfields like the sociology of science and culture to make contributions to other areas of sociology, including knowledge production and stratification. Medical education remains, therefore, a powerful window into social processes of broad sociological importance and will continue to be a driver of such foundational research in the future as the landscape of professional work continues to change. Put differently, medical education remains foundational for sociology because medical education represents both a microcosm of social life and because what happens in it directly shapes other areas, such as patient experiences and organizational dynamics in health care institutions.

Finally, we offer six potential future research directions: (1) inequalities in medical education, (2) socialization across the life course and new institutional forms of gatekeeping, (3) provider well-being, (4) globalization and medical education, (5) medical education as knowledge-based work, and (6) potential impacts of the COVID-19 pandemic on medical training. Our review serves as a call to action for both medical sociology and the larger discipline to further explore how power, socialization, and inequality among professionals matter for social dynamics in health and beyond.

## Professional Socialization

Studies of professional socialization, such as Becker et al. (1961) and Merton et al. (1957), formed the backbone of the sociology of medical education. Since that time, the three central themes in the contemporary literature on professional socialization have been professional identity formation, learning to do clinical work, and learning to interact in professional ways.

### Professional Identity Formation

Fundamentally, medical training entails a status transformation from the lay world to the medical world. As part of this process, medical training provides opportunities to experiment with professional roles in more and less realistic clinical settings, like the anatomy lab (Vinson 2020) or during third-year clerkships (Perrella et al. 2019). Although opportunities for this identity work and play (Vinson 2020) are provided to trainees by medical schools and faculty, and thus structured by the norms and values of the profession, trainees actively negotiate their relationship to them. In other words, crafting an identity is active work (Holloway 2014; Thomas 2018), which is not only dependent on using medical knowledge to assert professional status but also is shaped by peer cultures (Vinson 2019). This perspective on identity development represents the legacy of Becker et al.’s (1961) move away from functionalist assumptions that medical students simply adopt the norms and values of their profession and acknowledges the influence of the symbolic interactionist tradition in the sociology of medical education.

One important mechanism of professional reproduction is that trainees learn about their roles from more senior physicians who train them. Recent work in this vein has examined how attire reinforces status hierarchies among health professionals (Jenkins 2014) and how clinical teaching can encode certain subspecialties and procedures as heroic and masculine, increasing their prestige (Johannessen 2014). Trainees also observe senior physicians to learn about their roles vis-à-vis patients (Vinson 2016), future trainees (Jenkins 2020), other members of the profession (Brooks 2016), and even the state (Menchik 2012). These studies highlight how professional socialization is a reproductive process that preserves professional culture across generations of trainees, although a rising emphasis on small-group teaching means that professional socialization may occur in uneven or unstandardized ways (Olsen 2019). Faculty can be strong forces of socialization, and research continues to identify homogenizing effects of medical education that can undo the work of creating more diverse classes of medical students (Beagan 2000).

In investigating professional socialization as an actively negotiated process, scholars have focused on trainee responses to medical education. Trainee resistance can be identified in instances of curriculum change; for example, research has shown that the introduction of work-hour restrictions threatens the professional identity that surgical residents acquire during their training (Brooks and Bosk 2012; Coverdill et al. 2010; Kellogg 2011). Likewise, students are known to experience a significant decrease in empathy as they progress in their training, suggesting that students may “shed” empathy as a way of decreasing their vulnerability to stressors (Michalec 2010), such as being questioned by patients (Sointu 2017). Examining resistance can help scholars observe professional identity formation in action and learn about its effects on patient care. Indeed, recent research has shown that individuals’ actions in clinical work align with their attitudes about professional roles (Bochatay et al. 2017), pointing to the clinical impact of professional identity.

### Learning to Do Clinical Work

A prominent historical theme in the literature on professional socialization and learning to do clinical work is the process of managing uncertainty (Fox 1957). Contemporary research has demonstrated how managing uncertainty is shaped by the current clinical environment and embedded in distinct interpersonal and institutional contexts (Bochatay and Bajwa 2020). For example, Timmermans and Angell (2001) demonstrate that residents develop “evidence-based clinical judgment” as they learn to manage uncertainty in an ongoing fashion—meaning they negotiate styles of incorporating evidence-based knowledge into their decision-making.

Learning to do clinical work also highlights the relationship between skill development, emotion, and interpersonal relationships. Research shows that when learning high-stakes procedures, trust and reciprocity between trainees and supervisors matter for how mistakes are framed (i.e., as “permitted”; Shelton, Mort, and Smith 2018). Other studies demonstrate that inadequate preparation leads residents and junior doctors to order more tests, feel negatively toward patients, and feel stressed in their clinical encounters (Brooks et al. 2018). Such residents must also navigate delicate moral, professional, and institutional constraints, particularly in end-of-life care, which can sometimes lead them to defy patients’ wishes (Jenkins 2015). When it comes to managing overwhelming amounts of work, Szymczak and Bosk (2012) explore how residents develop efficiency not only as a strategy but also as a social norm. Overall, these studies help researchers understand how trainees actively respond to institutional and interpersonal constraints in their work.

### Learning to Interact in Professional Ways

Learning to do clinical work is also about adopting interactional norms, which are responsive to the larger social and political landscapes in which the profession is situated (Everitt et al. 2020). Indeed, how medical students learn to interact with patients reflects ongoing debates regarding medicine’s professional dominance and its centrality to social control. For example, Vinson (2016) shows how physicians use patient empowerment discourse as a countervailing power against patient consumerism to mitigate deprofessionalization. Underman (2020) further demonstrates that medical students learn ways of relating to patients that allow them to strategically uphold professional authority in the clinical encounter—a form of social control.

As a result, medical students today must learn to navigate the physician-patient relationship in new ways and through new methods that have not previously been documented by sociologists. As structural changes in health care place more demand on physicians to engage empathetically with patients, communication skills have become an increasingly intentional and intensive part of the medical curriculum (Underman and Hirshfield 2016). Vinson and Underman (2020), for example, use the lens of emotional labor to explore teaching clinical empathy and communication skills in contemporary medical education. These efforts reflect a particularly enduring tension between “learning to cure” and “learning to care” (Michalec 2011) or between scientific knowledge and humanist values. Sociologists have proposed numerous efforts to foster patient-centered communication skills in medical students, including increased formal teaching and testing of psychosocial skills (Michalec 2011). However, there is also evidence that undergraduate education matters significantly, raising questions about the typical pipeline for medical school admissions from STEM fields. Hirshfield, Yudkowsky, and Park (2019) found that medical students who majored in humanities or social science scored higher in communication skills exams, and Olsen and Gebremariam (2020) found that humanities and interpretive social sciences majors entered and left medical school with higher empathy than their STEM peers. All of these trends demonstrate challenges in the values of the profession as well as in the context of medical work.

Finally, new technologies shape the acquisition of communication skills in medical education (Pilnick et al. 2018). In the past 20 years, the largest change has been the use of standardized patients (SPs), or laypeople who are trained to role-play as patients, in teaching and assessment. Underman (2020) describes how concerns about communication skills training during the 1970s and 1980s led to widespread adoption of standardized communication skills tests. Training with SPs may present advantages; research has demonstrated that giving medical students control over the emotional intensity of SP encounters allows them to engage in difficult emotional tasks more comfortably (Lefroy, Brosnan, and Creavin 2011). Taken together, these new technologies for teaching communication skills align with enduring themes in medical sociology about the tension between scientific or objective ways of knowing and intersubjective experiences. They bring into question new contexts within which medicine’s drive toward science and standardization is expressed.

In sum, the past 20 years have actively engaged with, and extended, the discipline’s roots in the professional socialization of physicians. These recent studies demonstrate that professional identity formation is an active process that is responsive to status hierarchies, knowledge and uncertainty, and changing expectations of professional conduct. Still, professional socialization remains an important avenue for shaping trainee identity, enacting social control both through training experiences and later during clinical encounters. Such work is essential for the broader field of sociology to reexamine authority and professional role-taking as public’s trust in experts—such as physicians—has declined. Next, we consider changes in medical knowledge and how sociologists have understood these shifts.

## Knowledge Regimes

Much of medical sociology was built on a critique of the construction and organization of medical knowledge. With foundational concepts such as the clinical gaze (Foucault 1994), labeling (Scheff 1974), and (bio)medicalization (Clarke et al. 2003; Conrad and Schneider 1980; Zola 1972), sociologists have elucidated the dominant knowledge regime: the biomedical model. This model constitutes a body of knowledge and practices that prescribe symbols, language, socializing processes, and cultivated dispositions and impose order on the disordered natural world (Mishler 1981). The biomedical model has been critiqued for promoting a reductionism that purports neutrality when in actuality, empirical observations and diagnostic categories stem from a distinctly Western epistemological framework and research samples that tend to be white and middle-class (Epstein 2007).

The biomedical model structures medical education practices, methodologies, and research (Martimianakis and Albert 2013). Recently, however, sociologists have drawn attention to a new knowledge regime that has emerged in response to—and is necessarily shaped by—biomedicine: cultural competence. The values, practices, and identities within biomedicine rush, fragment, and standardize patients, creating a physician-patient interaction that reflects and reinforces power imbalances that disadvantage certain types of patients (Heritage and Maynard 2006). In response, the cultural competence knowledge regime claims that future physicians must learn how to transmit information, align goals, and reflect on their own biases (Betancourt 2006). Implicit bias training often falls under this knowledge regime.

Recent studies in this area have advanced the sociology of knowledge by elucidating the poorly understood process of knowledge translation in the social sciences and by countering previous research suggesting that social science cannot be instrumentalized to address social problems in the same way as “bench” science (Olsen 2020). These studies also reveal the limitations of this translational approach: Although the ideals undergirding the emergence of cultural competence and implicit bias regimes promise to ameliorate disparities faced by socially marginalized groups, their implementation has largely tended to reinforce biomedicine’s individualizing tendencies (Beagan 2003; Olsen 2020). Scholars have shown how these knowledge regimes tend to depict human difference as static and fixed rather than fluid (Hester 2015), emphasize geneticized or individualistic behaviors over structural and systemic processes (Metzl and Hansen 2014), and place patients as the focal point rather than reflecting on the profession itself (Fox 2005). To the latter point, Beagan (2003:613) notes the inadequacy of a curriculum that does not integrate power relationships in cultural competence training since “the experience of learning about ‘Others’ can be a type of voyeurism, stereotyping, exoticization, identifying the ‘deviant’ features of ‘those peoples’ [*sic*] lives.”

Issues with instruction on human difference can be further illustrated by contemporary studies of how medical schools approach race and LGBTQ topics. From overwhelmingly white- or light-skinned-centric content in textbooks (Louie and Wilkes 2018) to presenting biological conceptualizations of race (Braun and Saunders 2017) to assuming that a person’s social identity is the source of the peson’s professional skills (Michalec et al. 2017; Olsen 2019), the systemic nature of racism in the United States is not addressed by the cultural competence knowledge regime. Similarly, research has documented how little students learn about working with LGBTQ populations, particularly transgender patients (Beagan, Fredericks, and Bryson 2015), and how that, in itself, is a manifestation of heteronormativity in medical education (Giffort and Underman 2016; MacFife 2019; Murphy 2016). Further studies have shown how clinical faculty marginalize psychosocial skills as being outside their professional purview (Raz and Fadlon 2006), where the devaluation of cultural competence compared to biomedical knowledge demotes cultural competence training to “lower status” (Raz 2003).

As a result, many scholars have called for more critical social science in medical curricula (Kendall et al. 2018; Sales and Schlaff 2010). Others point out, however, that curricular reform may not dismantle existing power relationships in academic medicine and health care and that the curriculum—hidden or otherwise—does not generally address power and conflict (Michalec and Hafferty 2013; Olson and Brosnan 2017; Paradis and Whitehead 2018).

Thus, despite advocating for more complex understandings of culture in cultural competence (Powell Sears 2012), medical school curricula are left wanting (Constantinou et al. 2018). Although part of this may be the lingering effects of the biomedical model (Brosnan 2011), scholars have also argued that neoliberal pedagogical regimes may play a role. Specifically, Sointu (2020:853) argues that for medical students, the “allure of the more ‘downstream’ determinants of health lies in their alignment with neoliberal ideas of health, selfhood and the state.” This neoliberal logic even extends to the self-care regimes that have emerged for medical students themselves (Mitchell et al. 2016).

Indeed, this recent literature draws on classic themes in medical sociology about the biomedical model and the nexus of power and knowledge. It contributes to broader sociological conversations about the politics of knowledge production, raising crucial questions about the role of human difference in science and medicine, the dynamics of inclusion and exclusion of marginalized groups, and the centrality of critical or conflict perspectives to understanding professional work. We continue these themes next by exploring literature on professional stratification.

## Stratification In The Profession

Whereas early sociologists largely viewed medicine and other professions as “compan[ies] of equals” (Friedson and Rhea 1963:119), recent scholarship has emphasized how trainees are stratified by class, race-ethnicity, sex-gender, and training backgrounds. More than ever before, scholars have brought stratification to bear on medical education, thereby reinforcing ties between medical sociology and the larger discipline. They have also used medical education as a case to broaden sociological knowledge about stratification more generally.

### Class

Class differences between trainees have particularly caught sociologists’ attention in the past 20 years. The socioeconomic composition of U.S. allopathic medical schools has remained remarkably stable for the past three decades, with roughly three-quarters of matriculating students coming from the top two household-income quintiles (Youngclaus and Roskovensky 2018). This stability may be partly due to high tuition. However, recent research points to subtler forms of exclusion along class lines. For example, Grace (2017) finds that premedical students who identify as lower status are more likely to doubt their likelihood of going to medical school and to share concerns about letting down their communities.

These subtle, class-based processes continue into medical school, where students with lower socioeconomic status (SES) and first-generation college graduates experience “everyday classism” (Beagan 2005:777). Studies show that lower-SES and first-generation students in Canada, Australia, and the United Kingdom are often tacitly discouraged from pursuing medical school (Bassett et al. 2018; Southgate et al. 2017), experience ambivalence about their social mobility and the growing social distance between them and loved ones (Brosnan et al. 2016; Southgate et al. 2017), express financial concerns (Brosnan et al. 2016), and grapple with feelings of inadequacy (Bassett et al. 2018; Southgate et al. 2017). They also struggle with “playing the game” (Jenkins 2020:34–35), defined as not only having a “feel” for how to succeed in medicine (Bourdieu and Wacquant 1992) but also the process of gaining admission to medical school, residency, and beyond. Others note they lack an “inherited medical habitus” (Brosnan et al. 2016:847), or ease with which to navigate medical training (Brosnan 2009), including access to insider information, social networks, and the ability to “fit in” seamlessly (Beagan 2005). These patterns extend into residency, where physicians from lower-SES backgrounds, who are more likely to attend lower-status medical schools, have fewer resources to successfully play the game (Jenkins 2020). Thus, sociological work has begun identifying some of the more implicit barriers facing lower-SES medical trainees largely by incorporating and expanding insights from Bourdieu and others in the sociology of culture to help understand why socioeconomic diversity remains stagnant in medicine.

### Race-Ethnicity

Compared to class diversity, racial and ethnic diversity among medical trainees has slightly increased in recent years (Lett et al. 2019), but sociological studies suggest that students of color—particularly Black and Latinx students—remain highly marginalized. Students regularly experience overt forms of discrimination, like racist jokes and segregation, while also being confronted with more insidious insults, like widespread denial of racism in medical school (Beagan 2003), a lack of role models of color (Lempp and Seale 2004, 2006), and expectations that they will work in underserved communities after graduation (Michalec et al. 2017). When medical schools do teach about race/racism, many educators “conscript” students of color into teaching the material themselves, thereby perpetuating racism by burdening racial minority students and downplaying the importance of the subject (Olsen 2019). These processes can have important implications for physician career trajectories. Davis and Allison (2013) found that Black men medical students were more likely to enter high-prestige specialties than their white peers when controlling for differences in racial discrimination and mentorship, suggesting that racism and not personal preference contribute to racial inequality in specialization trends. Still, experiences of medical students—and educators—of color remain highly understudied (Olsen 2019) and represent an important area for continued research, particularly as the national conversation shifts toward addressing structural racism. There is even less work examining the intersections of gender, race, and/or class, and much remains unknown about how these axes of inequality interact with other medical hierarchies.

### Sex-Gender

Compared to race-ethnicity, sociologists over the past 20 years have paid closer attention to sex-gender inequality across the training life course, building on earlier work by Lorber (1984) and others. Although the number of women enrolling in medical school has now surpassed men (American Association of Medical Colleges 2017), women students experience less enjoyment, more stress, and more negative interactions with faculty during premedical coursework than men, which contributes to their greater likelihood of leaving the premed track (Grace 2019). Women medical students also experience more sexual harassment and gender-based discrimination than men despite increasing numbers of women in medicine. To explain this persistence, Hinze (2004) argues that harassment occurs within the context of a stable, rigid, and gendered hierarchical system that consistently puts women at the bottom, making mistreatment seem like a routine and even necessary part of medical training.

This mistreatment can have important consequences for women’s career trajectories. For example, German women medical students experience harassment three times more often than men and are thus more likely to rule out certain specialties, like surgery (Jendretzky et al. 2020). Unequal treatment persists into residency, where senior women trainees in emergency medicine are evaluated more critically than men with the same level of competence, pointing to a persistent and evolving bias against women, particularly as they progress through their training, because that bias did not exist among junior trainees (Brewer et al. 2020; Mueller et al. 2017). These recent studies have contributed to the broader sociological literature on gender inequality in the workplace. Brewer et al. (2020), for example, use role expectation theory to theorize why men trainees are deemed more competent than women trainees at the end of residency (but not at the beginning), thereby using medical education as a case to tackle a long-standing question in the sociology of gender: Why do women lag behind men at work when they do better than them in school? Studies like these make clear how the sociology of medical education is helping drive theoretical development in sociology more broadly.

### Inequalities between Training Contexts

In the past 20 years, sociologists have also investigated inequalities between educational institutions and their students. Contrary to earlier assumptions about homogeneity in medical education dating back to Freidson (1970), new research demonstrates important differences across organizations. Brosnan (2011), for example, finds a dichotomy in UK medical schools between more “scientific” approaches and more “clinical” approaches, with the former being viewed as more legitimate than the latter. Similarly, Jenkins (2018) illustrates how differences in resources between residency programs in American community hospitals compared with university hospitals can lead to unequal training, with potential implications for patients. Medical training has also globalized considerably in recent decades, with dozens of (largely) for-profit Caribbean medical schools opening during the early 2000s, raising questions about the quality of these institutions (Jenkins 2020). These findings have thus helped reorient sociological understandings of homogeneity within professions more broadly (Adams 2020).

Sociologists have also focused on status inequalities between students from different training backgrounds, including between American-trained MDs and osteopathic and international medical graduates (IMGs), who often train in segregated environments with negative consequences for trainees (Jenkins et al., 2020; Jenkins and Reddy 2016). Studies find, for example, that IMGs have special acculturation needs, such as unfamiliarity with shared decision-making (Osta et al. 2017), which may not necessarily be met in the low-resource training environments where IMGs typically train. Jenkins (2020) theorizes that such inequalities between trainees help medicine fulfill its “social contract” with U.S.-trained MDs, whereby in exchange for their hard work and dedication, they are near-guaranteed access to careers of their choice, whereas international and osteopathic graduates fill less desirable positions. Jenkins uses the case of medicine to arrive at a more general theory of “status separation” on the making of horizontal stratification among professionals—further evidence of how studies of medical education are contributing to broader sociological theory on stratification.

In sum, sociologists have addressed new topics in enduring questions about inequality in the professions. They have departed from some of the traditional assumptions of medical education research in the 1970s, when the profession was more homogenous, to account for a diversifying yet ever-hierarchical profession. They have notably incorporated theoretical insights from the sociology of culture, gender, and stratification to help understand inequality in the profession and, in so doing, have used medical education to expand those scholarly areas. We turn to associated questions of jurisdiction and boundary work next.

## Sociology Of The Field Of Medical Education

Some of the central questions that motivated early medical sociology focused on the structure of the profession and the proper jurisdiction of professional work (e.g., Freidson 1970). Sociologists since 2000 have begun to attend to larger-scale questions about medical education as a professional domain itself, interrogating who is authorized to do the work of teaching medical students (Underman 2020), what the nature of this professional work is, and what new questions of professional practice might arise in increasingly interprofessional clinical environments.

A key contribution by sociologists of medical education over the past 20 years has been to draw on insights from the sociology of science and knowledge to analyze and interrogate the larger field of medical education research itself, which extends far beyond the sociological “slice” of literature we review here. Medical education research emerged in the mid-1950s and crystallized quickly into a relatively institutionalized research field housed within medical schools (Kuper, Albert, and Hodges 2010). Sociologists have since identified several persistent tensions within the field. These tensions include conflict between the desire for “objective” tools and the recognition of the limitations of standardization (Paradis et al. 2017; Rangel et al. 2016; Underman 2020) and friction between scholars *of* medical education and practitioners *in* medical education (Albert 2004). This latter tension highlights an ongoing question among medical education scholars who note the speed with which the field has developed but also emphasize the ambiguity of its jurisdictional boundaries (Martimianakis and Albert 2013).

Most sociological scholarship describing medical education as a field has been critical, noting that interdisciplinary research in the area tends not to be theoretically informed and is sometimes viewed as low quality (Albert 2004; Albert, Hodges, and Regehr 2007; Albert and Reeves 2010; Brosnan 2010). Sociologists have made strides to encourage greater interprofessional collaboration with PhD-trained scholars (Albert et al. 2007) and have increased theoretical engagement by medical educators, particularly with sociological theory (Albert and Reeves 2010; Brosnan 2010; Frank 2013). For example, Brosnan (2009) used Bourdieu’s work to develop a comprehensive theory of medical education that bridged research on individual student outcomes and institutional research on medical education contexts. Scholars have also used medical education as a case to advance theory about interprofessional collaboration more broadly (Frickel, Albert, and Prainsack 2016).

At the same time as interprofessional collaboration between practitioners and social scientists has increased, so too has interprofessional education and collaboration within medical education contexts more broadly. Sociologists have noted many challenges faced by those developing interprofessional education (IPE) programs, including entrenched, often gendered, status hierarchies (Bell, Michalec, and Arenson 2014). Indeed, these hierarchies permeate not only the collaborations themselves but also the language that medical educators use to write about them (Paradis et al. 2017). Yet power and conflict are rarely at the center of interdisciplinary IPE research (Paradis and Whitehead 2018). Work that does highlight such power dynamics is often conducted by sociologists, like Oh’s (2014) ethnographic study of hospitalists, which focused on professional jurisdiction and boundary work within medical specialties. Notably, sociologists have provided evidence for the counterintuitive argument that IPE may in fact *increase* professional boundaries and hierarchical divides rather than foster collaborative relationships (Whyte et al. 2017).

In this way, the field of medical education has become an object of inquiry in recent sociological work, which incorporates new forms of knowledge production, types of experts, and kinds of interprofessional practice that now occur. These studies draw on and advance central themes in sociology more broadly about boundaries and jurisdiction in professional and expert work. They also contribute to the sociology of science on interdisciplinarity. Medical education *and* the study of medical education have therefore both served as objects of inquiry in the past 20 years.

## Future Directions

As we have shown, over the past 20 years, sociologists have extended classic threads of scholarship on medical education. Much of this new scholarship continues to approach medical education from a constructivist and interactionist lens and is qualitative, thereby extending the tradition that started in the 1950s to 1960s. Sociologists have also forged important new ground, however, by applying quintessentially sociological ideas about culture, knowledge, stratification, and professions to the study of medical education. In the process, they have enriched the substantive understanding of medical training *and* reinforced medical sociology’s significance to the broader discipline of sociology in areas like professional socialization, knowledge production, and stratification. But this work is far from over. Both medical sociology and the larger discipline ought to continue investigating how dynamics within medical education can matter for understanding broader social processes and how broader social processes matter for understanding medical education. Here, we outline six specific directions for future research.

### Inequalities in Medical Education

Future research should further consider how medical training reproduces social inequalities. On one hand, medical training is known to transmit stereotypes and other harmful beliefs about marginalized populations, a theme that beckons more sociological inquiry (Giffort and Underman 2016; Sointu 2017). Sointu (2017), for example, revealed how medical students learn ideas about “good” and “bad” patients that align with the extant social hierarchy. On the other hand, the incorporation of new types of knowledge and new populations could help ameliorate inequalities in health care. More research is therefore needed on the relationship between medical education and social inequalities, such as on how changing knowledge regimes like new “structural competence” curricula (Metzl and Hansen 2014) may exacerbate or palliate inequality. Other questions include: How do knowledge regimes capture and perpetuate health inequalities? What types of institutional gatekeeping exist to keep marginalized students out of medical school or sort them into lower-status specialties? The role of mentors and faculty in supporting or inhibiting marginalized students’ career aspirations also deserves more attention. Indeed, current organized responses to structural racism (e.g., White Coats for Black Lives) demand that researchers examine these issues within medical education (Yancy 2020).

### Socialization across the Career Path and New Forms of Institutional Gatekeeping

Future research should continue expanding the scope of medical training typically considered by sociologists and examine new institutions implicated in medical education. Sociologists have increasingly begun widening their gaze to more systematically include different parts of the career life course. For example, a number of recent studies have focused on premedical students (Grace 2018a, 2018b, 2021; Lin et al. 2014; Michalec and Keyes 2013; O’Connell and Gupta 2006; Olsen 2016). Given the popularity of preclinical college majors, this population, as well as their transition to medical school, is ripe for further study. As work on premedical burnout and attrition demonstrates, members of marginalized groups face more barriers to completion of preclinical programs and admission to medical school (Grace 2017, 2019).

Just as standardized tests and admissions serve as barriers for prospective medical students, comparable institutional gatekeeping exists in later stages of training in the form of medical board licensing exams and continuing medical education requirements. Indeed, medical education’s governing bodies are numerous and powerful and often have overlapping missions. Yet little sociological research has explored the role of these organizations in medical trainees’ (including premedical students) professional socialization, in (re)producing status hierarchies within and between the health professions, or in the development of the field of medical education. Such research is crucial because it highlights the relationships between medical education institutions and the individuals within them (Brosnan 2010). For instance, how might recent demands for the elimination of the clinical skills portion of the United States Medical Licensing Examination represent forms of resistance to institutional gatekeeping? And how might new maintenance of certification requirements be contributing to provider burnout?

### Provider Well-Being

Relatedly, another area that is ripe for sociological inquiry is professional mental health and well-being, particularly among trainees. Professional organizations have increasingly framed burnout as an “epidemic” among clinicians, and indeed the rates of mental illness and suicidal ideation are overwhelming (National Academies of Medicine 2019). This likely represents a structural-level—not individual-level—problem in the profession. But although research on physician well-being has exploded in recent years, this literature is predominantly in medicine and psychology and tends to focus on the problem’s *individual*-level causes and solutions. How do physicians interpret burnout’s causes and effects? How do the increasing burdens of evaluation, workload, and conflicting values shape educators’ well-being? How does educator well-being shape trainee well-being? And more broadly, what are the structural, professional, and organizational factors shaping physician well-being?

### Globalization and Medical Education

Another future direction to consider is the relationship between globalization and medical education. U.S. premedical students, medical trainees, and physicians are increasingly seeking international opportunities for training (Leng, McKinley, and Opalek 2018). These types of trips raise important questions about privilege, nationality, and “voluntourism” (Martimianakis and Hafferty 2013) as well as crucial questions about capitalism and colonialism (Bleakley, Brice, and Bligh 2008). Similarly, economic and political instability forces some physicians to leave home to seek safety and opportunities in other countries (Bell and Walkover 2020). In the United States, many immigrants and especially refugees face significant barriers to licensure, depending on their national origin (Walkover and Bell 2020). These global dynamics have not received much attention from sociologists. Likewise, the U.S. model of a central national licensing exam is somewhat unique (Price et al. 2018), although questions are being raised about the utility of licensing exams in other countries (Rizwan et al. 2018) as U.S.-style testing is increasingly implemented internationally. The effects and outcomes of translating the U.S. model into other cultural contexts is largely understudied, as are the organizational logics and dynamics behind such global flows of knowledge.

Thus, the globalization of medical education is a promising area for sociologists to investigate. How might experiences abroad, migrating or fleeing to work in the United States, or being educated in a United States model shape the identities, professional practices, and skill sets of diverse physicians? What happens to U.S.-based knowledge regimes as they cross borders or are acted upon by new social agents? What organizational interests—symbolic, social, or financial—are served through the global expansion of U.S. medical education?

### Medical Education as Knowledge-Based Work

Historically, the sociology of medical education developed alongside the sociology of professions. However, the disciplinary emphasis on sociology of professions has been displaced by an emphasis on knowledge-based work (Gorman and Sandefur 2011). Adopting this emphasis on knowledge-based work reflects recent developments in medical practice. For example, students and physicians are often trained to work alongside increasingly powerful and specialized health professionals like nurse practitioners and physician assistants, which has promoted interest in interprofessional education. In light of these developments, future sociological work on medical education should investigate the impact of new forms of training on professional identity formation, clinical team communication, and clinical knowledge production and legitimacy.

There is continued interest in new skills required by physicians, particularly those related to emotion and embodiment—areas that would be ripe for analysis by sociologists broadly interested in culture. More attention is necessary to understand how medical trainees develop the “feel” for embodied skills like palpation (Underman 2020), for example, as well as new styles of emotional expression and medical power in the clinical encounter (Timmermans 2020; Vinson 2016; Vinson and Underman 2020). Future work on emotion, embodiment, and medical power should continue to examine how physicians learn interactional styles. How is medical power expressed in contemporary clinical work? How might we understand the social organization and transmission of forms of feeling, sensation, and emotion?

### The Effects of the COVID-19 Pandemic

Finally, at the time of writing, the world is in the midst of the COVID-19 pandemic with limited strategies to mitigate its spread. The pandemic has exposed on a global stage enduring concerns among medical sociologists about health inequalities, such as higher mortality rates from the virus among Black and Latinx populations. Health professionals are overwhelmed and dying from the virus itself or from suicide. The mental health consequences of prolonged isolation and trauma loom large. Medical educators have scrambled to continue coursework and clinical rotations remotely. Likewise, licensing boards have had to rework both basic and applied knowledge testing. For sociologists of medical education, crucial questions arise: How will this pandemic shape who desires—and is able to—pursue a medical career? How will the economic impacts on hospitals and universities reshape medical training at all stages of the professional life course? How will training institutions redesign education to embrace new virtual technologies? And, indeed, how will social distancing measures impact medical sociologists’ research?

## Conclusion

The resurgence of interest in medical education over the last 20 years merits continued scholarship and momentum. Medical education was part of early medical sociology’s bread and butter—it was at the core of foundational scholarship in the subdiscipline and helped launch new lines of inquiry within broader sociology related to professions (Hall 1948), socialization (Becker et al. 1961; Merton et al. 1957), and social control (Bosk 1979). As our review demonstrates, the past 20 years have continued to develop those classic lines of inquiry by showing, for instance, how trainee resistance to professional socialization makes professional identity formation an active process and by complicating previous understandings of homogeneity in the profession. It has also shown how recent research has helped forge new lines of inquiry in the areas of knowledge production and stratification, thus deepening the ties between medical and general sociology.

Indeed, in the past 20 years, we have seen enduring questions about the nature of professions—their boundaries, their power over their members and the public, and their internal stratification—as well as recent developments in sociological theory on expertise and knowledge-based work evident in studies of the sociology of medical education. These insights have been inflected with cutting-edge sociological work on gender, race, and other forms of inequality. We also see the continued renewal of foundational themes like role-taking and new work in symbolic interactionism on learning and resistance. Foundational work on professional authority and the nexus of power and knowledge in medicine is also being updated with new insights about how expertise rearticulates its social and cultural power in the face of social protest and transformation in its economic and institutional bases. In all of these ways, other subfields of sociology, such as the sociology of science and culture, are influential for studies of medical education—and vice versa. We therefore see opportunities for sociologists in other areas, like the sociology of culture, to leverage insights from the sociology of medical education on topics like emotions, embodiment, and inequality in organizational contexts.

Given the broad themes in the existing literature that we review here and our calls for future research, the sociology of medical education is timelier than ever. As the world grapples with the structural and personal challenges of the COVID-19 pandemic and its profound effects, the sociology of medical education stands ready to contribute new theoretical and methodological insights of relevance to the discipline as a whole.
